# Short-term outcomes of robot-assisted versus conventional laparoscopic surgery for mid and low rectal cancer after neoadjuvant chemoradiotherapy: a propensity score-matched analysis

**DOI:** 10.1007/s11701-022-01498-3

**Published:** 2022-11-21

**Authors:** Takahiro Yamanashi, Hirohisa Miura, Toshimichi Tanaka, Akiko Watanabe, Keigo Yokoi, Ken Kojo, Masahiro Niihara, Keishi Yamashita, Takeo Sato, Yusuke Kumamoto, Naoki Hiki, Takeshi Naitoh

**Affiliations:** 1 Department of Lower Gastrointestinal Surgery, Kitasato University School of Medicine, 1-15-1 Kitasato, Minami-Ku, Sagamihara, Kanagawa 252-0374 Japan; 2 Department of Upper Gastrointestinal Surgery, Kitasato University School of Medicine, 1-15-1 Kitasato, Minami-Ku, Sagamihara, Kanagawa 252-0374 Japan; 3 Division of Advanced Surgical Oncology Department of Research and Development Center for New Medical Frontiers, Kitasato University School of Medicine, 1-15-1 Kitasato, Minami-Ku, Sagamihara, Kanagawa 252-0374 Japan; 4 Research and Development Center for Medical Education, Department Clinical Skills Education, Kitasato University School of Medicine, 1-15-1 Kitasato, Minami-Ku, Sagamihara, Kanagawa 252-0374 Japan; 5 Department of General, Pediatric and Hepatobiliary-Pancreatic Surgery, Kitasato University School of Medicine, 1-15-1 Kitasato, Minami-Ku, Sagamihara, Kanagawa 252-0374 Japan

**Keywords:** Conventional laparoscopic surgery, Neoadjuvant chemoradiotherapy, Rectal cancer, Robot-assisted laparoscopic surgery, Short-term outcomes

## Abstract

The benefits of robot-assisted laparoscopic surgery (RALS) for rectal cancer remain controversial. Only a few studies have evaluated the safety and feasibility of RALS following neoadjuvant chemoradiotherapy (NCRT). This study aimed to compare the short-term outcomes of RALS versus conventional laparoscopic surgery (CLS) after NCRT for rectal cancer. Propensity score matching of 111 consecutive patients who underwent RALS or CLS after NCRT for rectal adenocarcinoma between February 2014 and February 2022 was performed. Among them, 60 matched patients were enrolled and their short-term outcomes were compared. Although operative time, conversion rate to open laparotomy and blood loss were comparable, the incidence of postoperative complications, including anastomotic leakage, was significantly lower, urinary retention tended to be lower, and the days to soft diet intake and postoperative hospital stay were significantly shorter in the RALS than the CLS group. No postoperative mortality was observed in either group, and there were no significant differences in terms of resection margins and number of lymph nodes dissected. RALS after NCRT for rectal cancer is safe and technically feasible, and has acceptable short-term outcomes. Further studies are required for validation of the long-term oncological outcomes.

## Introduction

Minimally invasive surgery (MIS) for rectal cancer has become popular in various types of surgeries. Evolution of surgical techniques and the introduction of more advanced instruments for rectal cancer have resulted in several advantages of MIS compared with open surgery, such as less blood loss, faster bowel recovery, and shorter hospital stay [1, 2]. Large randomized clinical trials (RCTs) have shown that conventional laparoscopic surgery (CLS) for rectal cancer demonstrated similar or better short-term outcomes compared with open surgery [1, 2], and similar long-term oncological outcomes as open surgery [3, 4]. In terms of operating in the narrow and deep pelvis, however, CLS for rectal cancer has several drawbacks, including inflexible instruments, uncomfortable non-ergonomic positions, poor visualization caused by an unstable camera, and inadequate traction by the assistant. This is reflected in the high conversion to laparotomy rate of 9–16% with CLS for rectal cancer, as reported previously [2, 5, 6]. Moreover, two large RCTs that assessed pathological outcomes with regard to circumferential resection margin (CRM) and total mesorectal excision (TME) completeness, indicating adequate surgical resection, revealed high positive CRM rates in CLS as compared to open surgery for rectal surgery, which might be related to the technical difficulty of laparoscopic surgery in the deep pelvis [5, 6].

Neoadjuvant chemoradiotherapy (NCRT) has been reported as a multimodal therapy for T3, T4, or N-positive mid and low rectal cancer, to reduce the local recurrence rate and increase the sphincter preservation rate [7–9]. However, MIS for rectal cancer after NCRT is challenging, and its post-treatment effects, such as tissue fibrosis and edema, further contribute to difficulty of the CLS procedure.

Robot-assisted laparoscopic surgery (RALS) for rectal cancer is one of the most recent advances in MIS. RALS has the potential to overcome the limitations of CLS, providing articulated instruments, improved ergonomics, a stable three-dimensional view, enhanced dexterity with tremor filtration, and motion scaling. Although the superiority of RALS versus CLS in terms of surgery conversion rates was not established in the ROLARR randomized clinical trial [10], several retrospective case–control studies [11–14] and small randomized clinical trials [15, 16] have reported favorable outcomes in terms of the safety and technical feasibility of RALS for rectal cancer. However, only a few reports comparing RALS and CLS have focused on short-term outcomes in patients with unfavorable preoperative clinical characteristics, such as receiving NCRT [17, 18]. We therefore designed a study to compare short-term outcomes of RALS and CLS after NCRT for mid and low rectal cancer.

## Methods

### Patients and data source

**Fig. 1:**
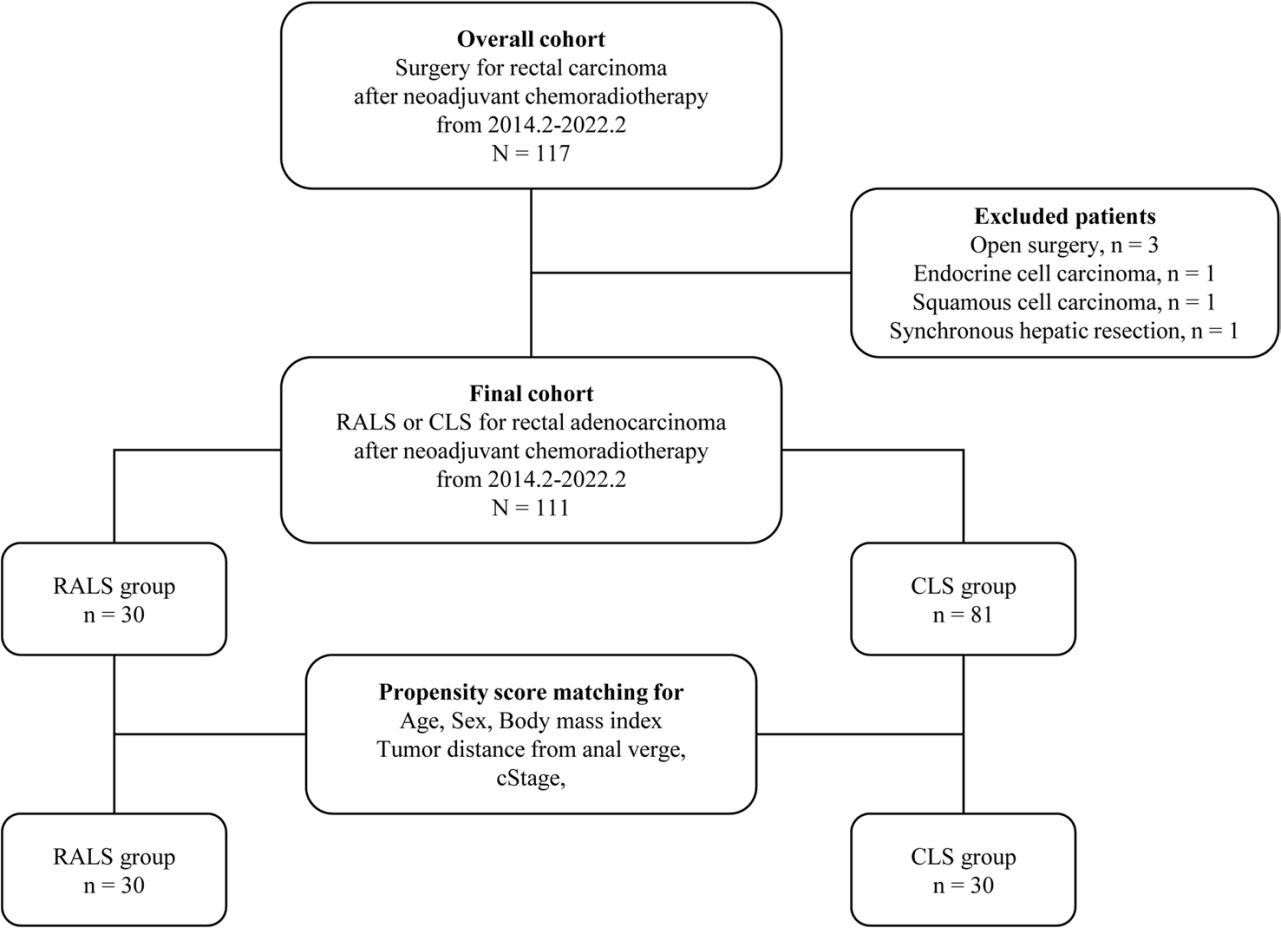
Study flow chart

This single-center, non-randomized, retrospective study compared RALS with CLS for low anterior resection (LAR) and abdominoperineal resection (APR) in rectal cancer patients. Patients who had histopathologically proven, primary mid and low rectal cancer with a clinical stage of cT3-4 or N-positive according to the Union for International Cancer Control (UICC) Tumor-Node-Metastasis (TNM) Classification, 8th edition [19], were operated on after receiving NCRT. Tumors in the mid and low rectum were defined as those located proximal and distal to the peritoneal reflection, respectively, based on barium enema examination or colonoscopy. Between February 2014 and February 2022, we retrospectively analyzed 111 consecutive rectal cancer patients who underwent RALS or CLS after receiving NCRT at Kitasato University Hospital, Japan. Thirty patients underwent RALS and 81 patients underwent CLS (Fig. 1). The choice of surgical technique depended on the surgeons’ discretion, schedule, or the availability of the Da Vinci surgical system (Intuitive Surgical Inc., Sunnyvale, CA).

Data used in this study were retrospectively obtained from our hospital medical records. The treatment effect of NCRT was classified based on the degree of degeneration or necrosis and fusion of cancer cells. No response was assigned a grade of 0, and a complete response was assigned a grade of 3, according to the Histopathological Response Criteria of the General rules for Clinical and Pathological Studies on Cancer of the Colon, Rectum and Anus edited by the Japanese Society for Cancer of the Colon and Rectum [20]. There were no missing data for the examined variables.

The protocol of this study was approved by our institutional review board (approval number B21-071) and conformed with the provisions of the Declaration of Helsinki. All patients were provided detailed information about the surgical procedure and provided their written consent.

### Neoadjuvant chemoradiotherapy and perioperative management

NCRT was administered according to our previously reported institutional guidelines [9, 21]. The radiotherapy regimen was in fractions of 1.8 Gy/day, given 5 days per week for 5 weeks. The total dose of radiation was 45 Gy. The field of treatment for radiotherapy was defined as the planned target volume which allowed for setup error, organ movement, and a 1 cm circumference around both the primary tumor and the adjacent enlarged lymph nodes, determined using computed tomography (CT). Tegafur/gimeracil/oteracil (S-1) (80 mg/m^2^/day) was given orally twice daily on days 1–5, 8–12, 22–26, and 29–33. Irinotecan (60–80 mg/m^2^/day) was given as an intravenous infusion on days 1, 8, 22 and 29.

Before and after administering NCRT, staging workup was performed using chest-abdominopelvic CT, pelvic magnetic resonance imaging (MRI), colonoscopy, and barium enema examination. Surgery was performed 8–10 weeks after NCRT completion. None of the patients underwent intersphincteric resection or Hartmann’s procedure after NCRT. Postoperatively, a liquid diet was started using a protocol such as enhanced recovery after surgery on day 1 morning in principal, oral intake to soft diet was allowed after the return of bowel movement.

### Operative procedure

RALS was performed by two certified surgeons using the Da Vinci Si or Xi surgical system (Intuitive Surgical Inc., Sunnyvale, CA) as a six- or five-port system. Among 30 patients who underwent RALS, the Da Vinci Si and Xi surgical system were used in 1 patient and in 29 patients, respectively. CLS used the five-port approach. Patients were placed in the lithotomy position with the head down at 15–20° and the right side down at 15°. All procedures were performed using a systematic approach, including colonic and pelvic phases in the robotic approach. The first phase (colonic phase) comprised inferior mesenteric artery and vein ligation and left-sigmoid mesocolon mobilization, and the second phase (pelvic phase) comprised pelvic dissection using TME principles. In patients who underwent LAR, the distal rectum was intracorporeally divided with a linear articulated endo-stapler loaded with a 45 mm or 60 mm cartridge. Bowel continuity was intracorporeally restored using the double stapling technique with 25 mm circular staples. All patients in both groups underwent standard curative resection with en bloc regional lymphadenectomy. Lateral lymph node dissection (LLND) was performed, as required, when the short diameter of lateral lymph nodes was larger than 7 mm on pre-CRT CT or MRI. Lateral lymph nodes were removed around the common iliac vessel, internal iliac vessel and obturator space, in the fat tissue outside the pelvic plexus, preserving all autonomic nerves. A diverting ileostomy was performed for all patients who underwent LAR.

### Outcome parameters

In this study, the outcome parameters were conversion rate to open laparotomy, operative time, blood loss, days to soft diet, postoperative hospital stay, postoperative complications, reoperation, postoperative mortality, proximal margin (PM), distal margin (DM), positive radial margin (RM), and number of lymph nodes dissected.

Conversion from laparoscopic surgery to open laparotomy was defined as the intentional extension of the laparoscopic incision beyond the incision necessary for specimen retrieval. Operative time was defined as the time between the initial skin incision and completion of wound closure. Postoperative complications, reoperation and mortality were defined as such events occurring during the postoperative hospital stay or within 30 days after surgery. Postoperative complications were categorized according to the Clavien–Dindo (CD) classification [22]. All such events were assessed by clinicians and documented in the database. The pathological parameters of the surgical specimens, including PM, DM, RM and the number of lymph nodes harvested, were recorded to assess the quality of surgery.

### Statistical analysis

Descriptive data are presented as the mean and standard deviation (SD) or median and range for continuous variables, and as the number of patients and percentage for categorical variables. Prior to propensity score matching, Student’s *t* test or Mann–Whitney *U* test was used for continuous variables, and the Chi-squared test was used for categorical variables. *P* < 0.05 was considered statistically significant. Propensity score matching was subsequently applied to minimize the possibility of selection bias and to adjust for differences in the baseline characteristics of patients. First, propensity score was obtained using multivariate logistic regression analysis. For calculating the propensity score, the following five covariates that might affect technical difficulties in surgery for rectal cancer were included in the model: age, sex, body mass index (BMI), tumor distance from the anal verge, and clinical stage. The next step was 1:1 matching using the caliper coefficient set at 0.2. This propensity score matching was applied to evaluate the effect of RALS on operative and pathological outcomes. Baseline characteristics, including covariates not entered into the propensity score model, operative results, postoperative complications, and pathological findings were subsequently compared between RALS and CLS. All statistical analyses were performed using statistical software JMP pro version 14 (SAS Institute Inc., Cary, NC).

## Results

A flow chart of this study is shown in Fig. 1. Of the 117 consecutive patients who underwent elective surgery for rectal cancer after receiving NCRT, three patients who underwent open surgery, two patients with histological confirmation of cancer other than adenocarcinoma, and one patient who underwent synchronous hepatic resection were excluded. Among the remaining 111 patients who underwent MIS, 30 patients (27.0%) underwent RALS (RALS group) and 81 patients (73.0%) underwent CLS (CLS group). The baseline characteristics of the overall cohort are listed in Table 1. Before matching, a tendency for differences between RALS and CLS group patients was observed with regard to sex (*p* = 0.0796) and tumor distance from the anal verge (*p* = 0.0712). After matching, 30 matched pairs were selected. The demographics of the propensity score-matched patients are listed in Table 1. The RALS and CLS groups were comparable with regard to baseline characteristics.

**Table 1:** Baseline characteristics before and after matching

Characteristics	Overall cohort (*N* = 111)			Propensity score-matched pairs (*N* = 60)		
	RALS group (*n* = 30)	CLS group (*n* = 81)	*p*	RALS group (*n* = 30)	CLS group (*n* = 30)	*p*
	*n* (%) or mean ± SD or median [range]			*n* (%) or mean ± SD or median [range]		
Age, years	62.4 ± 11.0	62.5 ± 10.2	0.9697	62.4 ± 11.0	61.7 ± 10.3	0.7913
Sex			0.0796			0.4872
Male	24 (80.0)	51 (63.0)		24 (80.0)	26 (86.7)	
Female	6 (20.0)	30 (37.0)		6 (20.0)	4 (13.3)	
Body mass index, kg/m^2^	22.4 ± 2.7	23.2 ± 4.1	0.3186	22.4 ± 2.7	22.7 ± 3.1	0.6610
ASA score			0.9168			0.4586
1	7 (23.3)	16 (19.8)		7 (23.3)	7 (23.3)	
2	21 (70.0)	59 (72.8)		21 (70.0)	18 (60.0)	
3	2 (6.7)	6 (7.4)		2 (6.7)	5 (16.7)	
Pre-CRT CEA level, ng/ml	7.4 [0.7–57.8]	4.8 [0.3–768.0]	0.1592	7.4 [0.7–57.8]	4.7 [1.0–768.0]	0.5945
Preoperative CEA level, ng/ml	2.8 [0.4–111.0]	2.3 [0.4–104]	0.1043	2.8 [0.4–111.0]	2.7 [0.7–104.0]	0.5104
Tumor distance from anal verge, mm	46.1 ± 16.4	52.6 ± 16.9	0.0712	46.1 ± 16.4	47.4 ± 16.5	0.7663
Tumor location			0.5107			1.0000
Mid rectum^a^	4 (13.3)	15 (18.5)		4 (13.3)	4 (13.3)	
Lower rectum^b^	26 (86.7)	66 (81.5)		26 (86.7)	26 (86.7)	
cT category			0.1678			0.5178
T3	23 (76.7)	71 (87.7)		23 (76.7)	25 (83.3)	
T4	7 (23.3)	10 (12.4)		7 (23.3)	5 (16.7)	
cN category			0.4897			0.8335
N0	17 (56.7)	36 (44.4)		17 (56.7)	16 (53.3)	
N1	8 (26.7)	30 (37.0)		8 (26.7)	10 (33.3)	
N2	5 (16.7)	15 (18.5)		5 (16.7)	4 (13.3)	
cM category			n.a			n.a
M0	30 (100.0)	81 (100.0)		30 (100.0)	30 (100.0)	
M1	0 (0.0)	0 (0.0)		0 (0.0)	0 (0.0)	
cStage^c^			0.2521			0.7952
II	17 (56.7)	36 (44.4)		17 (56.7)	16 (53.3)	
III	13 (43.3)	45 (55.6)		13 (43.3)	14 (46.7)	
ycT category			0.1723			0.4676
T1	2 (6.7)	3 (3.7)		2 (6.7)	1 (3.3)	
T2	13 (43.3)	20 (24.7)		13 (43.3)	9 (30.0)	
T3	13 (43.3)	54 (66.7)		13 (43.3)	19 (63.3)	
T4	2 (6.7)	4 (4.9)		2 (6.7)	1 (3.3)	
ycN category			0.3479			0.6584
N0	20 (66.7)	57 (70.4)		20 (66.7)	23 (76.7)	
N1	8 (26.7)	23 (28.4)		8 (26.7)	6 (20.0)	
N2	2 (6.7)	1 (1.2)		2 (6.7)	1 (3.3)	
ycM category			0.1462			0.5500
M0	28 (93.3)	80 (98.8)		28 (93.3)	29 (96.7)	
M1	2 (6.7)	1 (1.2)		2 (6.7)	1 (3.3)	
ycStage^d^			0.1117			0.5220
I	11 (36.7)	20 (24.7)		11 (36.7)	10 (33.3)	
II	7 (23.3)	36 (44.4)		7 (23.3)	12 (40.0)	
III	10 (33.3)	24 (29.6)		10 (33.3)	7 (23.3)	
IV	2 (6.7)	1 (1.2)		2 (6.7)	1 (3.3)	

^a^A mid rectal tumor was defined as one in which the lower border of the tumor was located proximal to the peritoneal reflection

^b^A low rectal tumor was defined as one in which the lower border of the tumor was located distal to the peritoneal reflection

^c^Pre-CRT clinical stage, TNM classification of malignant tumors, eighth edition

^d^Post-CRT clinical stage, TNM classification of malignant tumors, eighth edition

*RALS* robot-assisted laparoscopic surgery, *CLS* conventional laparoscopic surgery, *ASA* American Society of Anesthesiologists, *CRT* chemoradiotherapy, *CEA* carcinoembryonic antigen, *n.a* not applicable

The operative results for the overall cohort (*N* = 111) and propensity score-matched cohort (*N* = 60) are listed in Table 2. In the matched cohort, there were no conversions to open laparotomy in the RALS group and one (3.3%) conversion to open laparotomy in the CLS group. There were, thus, no significant differences in the conversion rates to open laparotomy between groups. In the matched cohort, median operative time was 365 min in the RALS group and 351 min in the CLS group, respectively. Thus, in the matched cohort, median operative time was comparable between the RALS and CLS groups (*p* = 0.3328). For both cohorts, the median days to soft diet were one day in the RALS group and three days in the CLS group, indicating significantly fewer days to soft diet in the RALS than the CLS group for both cohorts (*p* < 0.0001). The median durations of postoperative hospital stay were respectively nine and 12 days in RALS and CLS groups in the matched cohort, indicating a significantly shorter postoperative hospital stay in the RALS than the CLS group (*p* = 0.0011). In terms of intraoperative blood flow testing using the indocyanine green (ICG) test, which was only analyzed in patients who underwent LAR, the number of patients in whom the ICG blood test was performed was significantly higher in the RALS than the CLS group (*p* < 0.0001). For both cohorts, type of operation, LLND, blood loss, and combined resection were comparable between groups. None of the study subjects required blood transfusions.

**Table 2:** Operative results before and after matching

Characteristics	Overall cohort (*N* = 111)			Propensity score-matched pairs (*N* = 60)		
	RALS group (*n* = 30)	CLS group (*n* = 81)	*p*	RALS group (*n* = 30)	CLS group (*n* = 30)	*p*
	*n* (%) or median [range]			*n* (%) or median [range]		
Type of operation			0.8896			0.1063
Low anterior resection	14 (46.7)	39 (48.1)		14 (46.7)	8 (26.7)	
Abdominoperineal resection	16 (53.3)	42 (51.9)		16 (53.3)	22 (73.3)	
Lateral lymph node dissection	4 (13.3)	19 (23.5)	0.2263	4 (13.3)	7 (23.3)	0.3143
Diverting ileostomy	14 (100.0^a^)	39 (100.0^a^)	n.a	14 (100.0^a^)	8 (100.0^a^)	n.a
Blood flow testing using ICG	14 (100.0^a^)	4 (10.3^a^)	< 0.0001	14 (100.0^a^)	0 (0.0^a^)	< 0.0001
Operative time, min	365 [237–692]	341 [190–732]	0.1155	365 [237–692]	351 [210–732]	0.3328
Blood loss, ml	103 [5–627]	73 [5–1275]	0.5722	103 [5–627]	124 [5–1275]	0.4321
Transfusion	0 (0.0)	0 (0.0)	n.a	0 (0.0)	0 (0.0)	n.a
Conversion to laparotomy	0 (0.0)	1 (1.2)	0.4261	0 (0.0)	1 (3.3)	0.2362
Combined resection	2 (6.7)	5 (6.2)	0.9247	2 (6.7)	1 (3.3)	0.5500
Time to soft diet, days	1 [1-1]	3 [1-28]	< 0.0001	1 [1-1]	3 [1-28]	< 0.0001
Postoperative hospital stay, days	9 [7–51]	12 [7-35]	0.0005	9 [7–51]	12 [8-35]	0.0011

^a^Data were analyzed in patients with low anterior resection

*RALS* robot-assisted laparoscopic surgery, *CLS* conventional laparoscopic surgery, *n.a* not applicable, *ICG* indocyanine green

Comparison of the postoperative complications is shown in Table 3. The rates of moderate or severe complications (CD grade ≥ II) were significantly fewer in the RALS (10.0%) than the CLS group (33.3%) for both overall and matched cohorts (*p* = 0.0085 and *p* = 0.0250, respectively). There were no anastomotic leakages (which were only analyzed in patients who underwent LAR) > CD grade II in the RALS group. The rate of anastomotic leakage was significantly lower in the RALS group than the CLS group in both overall and matched cohorts (*p* = 0.0201 and *p* = 0.0358, respectively). There were also no cases of urinary retention > CD grade I that required placement of a urinary catheter in the RALS group. The rate of urinary retention tended to be lower in the RALS than the CLS group for both cohorts (*p* = 0.0720 and *p* = 0.0919, respectively). Only one patient required reoperation in each group, indicating no significant differences in reoperation rates between groups. The reasons for reoperation were ureter injury due to blind manipulations of the robotic arm in the RALS group and small bowel obstruction with adhesions in the CLS group. There were no postoperative mortalities in either group.

**Table 3:** Postoperative complications before and after matching

	Overall cohort (*N* = 111)			Propensity score-matched pairs (*N* = 60)		
	RALS group (*n* = 30)	CLS group (*n* = 81)	*p*	RALS group (*n* = 30)	CLS group (*n* = 30)	*p*
Characteristics	*n* (%)			*n* (%)		
Patient number, CD classification ≥ Grade II	3 (10.0)	27 (33.3)	0.0085	3 (10.0)	10 (33.3)	0.0250
Anastomotic leakage	0 (0.0^a^)	8 (20.5^a^)	0.0201	0 (0.0^a^)	2 (25.0^a^)	0.0358
Small bowel obstruction	1 (3.3)	5 (6.2)	0.5380	1 (3.3)	2 (6.7)	0.5500
Wound infection	2 (6.7)	9 (11.1)	0.4703	2 (6.7)	5 (16.7)	0.2210
Urinary retention	0 (0.0)^b^	5 (6.2)^b^	0.0720	0 (0.0)^b^	2 (6.7)^b^	0.0919
Pelvic abscess	0 (0.0)	2 (2.5)	0.2590	0 (0.0)	1 (3.3)	0.2362
Ureter injury	1 (3.3)	0 (0.0)	0.1041	1 (3.3)	0 (0.0)	0.2362
Urethral injury	0 (0.0)	1 (1.2)	0.4261	0 (0.0)	0 (0.0)	n.a
Diarrhea	0 (0.0)	1 (1.2)	0.4261	0 (0.0)	0 (0.0)	n.a
Gastric ulcer	0 (0.0)	1 (1.2)	0.4261	0 (0.0)	0 (0.0)	n.a
Bleeding	0 (0.0)	0 (0.0)	n.a	0 (0.0)	0 (0.0)	n.a
Pneumonia	0 (0.0)	0 (0.0)	n.a	0 (0.0)	0 (0.0)	n.a
CD classification ≥ Grade III	2 (6.7)	8 (9.9)	0.5894	2 (6.7)	3 (10.0)	0.6394
Reoperation	1 (3.3)	1 (1.2)	0.4866	1 (3.3)	1 (3.3)	1.0000
30-day postoperative mortality	0 (0.0)	0 (0.0)	n.a	0 (0.0)	0 (0.0)	n.a

^a^Data were analyzed in patients with low anterior resection

^b^Data were analyzed in patients who had urinary retention with CD grade ≥ I

*RALS* robot-assisted laparoscopic surgery, *CLS* conventional laparoscopic surgery, *CD* Clavien-Dindo, *n.a* not applicable

The pathological findings of the study subjects are listed in Table 4. No significant differences in terms of PM and DM were observed between RALS and CLS groups in the overall and matched cohorts. Positive PMs and DMs were not found in any patient. There were no significant differences in terms of positive RMs between the two groups in both cohorts, nor were there any significant differences in terms of the number of lymph nodes harvested in both groups. A significantly greater positive vascular invasion rate was observed in the CLS compared with the RALS group in the matched cohort (*p* = 0.0185). Tumor size, histological grade, lymphatic invasion, pathological stage, and treatment effect were similar between the two groups.

**Table 4:** Pathology findings before and after matching

Characteristics	Overall cohort (*N* = 111)			Propensity score-matched pairs (*N* = 60)		
	RALS group (*n* = 30)	CLS group (*n* = 81)	*p*	RALS group (*n* = 30)	CLS group (*n* = 30)	*p*
	*n* (%) or mean ± SD or median [range]			*n* (%) or mean ± SD or median [range]		
Tumor size, mm	29 [5–71]	27 [3–120]	0.6397	29 [5–71]	36 [5–105]	0.2798
Histological grade			0.6631			0.6394
G1-2 (pap/tub)^a^	27 (90.0)	75 (92.6)		27 (90.0)	28 (93.3)	
G3 (muc/por/sig)^b^	3 (10.0)	6 (7.4)		3 (10.0)	2 (6.7)	
Lymphatic invasion			0.4230			0.7174
Present	4 (13.3)	16 (19.8)		4 (13.3)	5 (16.7)	
Absent	26 (86.7)	65 (80.2)		26 (86.7)	25 (83.3)	
Vascular invasion			0.0377			0.0185
Present	9 (30.0)	42 (51.9)		9 (30.0)	18 (60.0)	
Absent	21 (70.0)	39 (48.1)		21 (70.0)	12 (40.0)	
Proximal margin, mm	178.3 ± 61.6	186.6 ± 51.2	0.4740	178.3 ± 61.6	201.9 ± 57.2	0.1289
Distal margin, mm	34.7 ± 16.1	32.1 ± 18.2	0.4978	34.7 ± 16.1	35.6 ± 15.8	0.8279
Positive radial margin	1 (3.3)	4 (4.9)	0.7091	1 (3.3)	1 (3.3)	1.0000
Number of lymph nodes dissected	13.7 ± 9.0	11.4 ± 7.7	0.1771	13.7 ± 9.0	12.3 ± 7.0	0.5045
yp T category			0.4721			0.9006
T0 / Tis	4 (13.3)	11 (13.6)		4 (13.3)	3 (10.0)	
T1	4 (13.3)	5 (6.2)		4 (13.3)	3 (10.0)	
T2	4 (13.3)	22 (27.2)		4 (13.3)	6 (20.0)	
T3	16 (53.3)	37 (45.7)		16 (53.3)	17 (56.7)	
T4	2 (6.7)	6 (7.4)		2 (6.7)	1 (3.3)	
yp N category			0.9254			0.4860
N0	21 (70.0)	54 (66.7)		21 (70.0)	18 (60.0)	
N1	6 (20.0)	19 (23.5)		6 (20.0)	10 (33.3)	
N2	3 (10.0)	8 (9.9)		3 (10.0)	2 (6.7)	
yc/yp M category			0.0414			0.2904
M0	27 (90.0)	80 (98.8)		27 (90.0)	29 (96.7)	
M1	3 (10.0)	1 (1.2)		3 (10.0)	1 (3.3)	
yp Stage^c^			0.2729			0.6420
0 / I	9 (30.0)	19 (23.5)		9 (30.0)	6 (20.0)	
II	8 (26.7)	26 (32.1)		8 (26.7)	9 (30.0)	
III	8 (26.7)	27 (33.3)		8 (26.7)	12 (40.0)	
IV	3 (10.0)	1 (1.2)		3 (10.0)	1 (3.3)	
pCR	2 (6.7)	8 (9.9)		2 (6.7)	2 (6.7)	
Treatment effect			0.9823			0.9602
Grade 1	17 (56.7)	46 (56.8)		17 (56.7)	18 (60.0)	
Grade 2	10 (33.3)	26 (32.1)		10 (33.3)	9 (30.0)	
Grade 3	3 (10.0)	9 (11.1)		3 (10.0)	3 (10.0)	

^a^Papillary adenocarcinoma/Well or moderately differentiated tubular adenocarcinoma

^b^Mucinous adenocarcinoma/Poorly differentiated adenocarcinoma/Signet-ring cell carcinoma

^c^Post-CRT pathological stage, TNM classification of malignant tumors, eighth edition

*RALS* robot-assisted laparoscopic surgery, *CLS* conventional laparoscopic surgery, *pCR* pathological complete response

## Discussion

Previous RCTs have demonstrated that the short-term and long-term oncological outcomes of CLS for rectal cancer are comparable to those of open surgery [1–4]. With the advent of RALS as the latest MIS, studies have been conducted to evaluate whether its application can overcome the limitations of CLS for rectal cancer. Many studies have shown that the surgical outcomes of RALS are comparable to those of CLS [10–12, 15–18, 23–29], although the proportion of patients receiving NCRT in those studies were variable and relatively low [11, 23]. In the present study, we evaluated the potential advantages of RALS after NCRT for rectal cancer by comparing operative and pathological outcomes between RALS and CLS in rectal cancer patients. The fact that we performed propensity score-matched analysis to obviate the possibility of selection bias and to adjust for significant differences in baseline characteristics of rectal cancer patients is a strength of our study.

The conversion rate to laparotomy of MIS reflects its technical complexity. Achieving a low conversion to open laparotomy rate is clinically important, because patients converted to open laparotomy are more likely to develop postoperative complications and local recurrence [30, 31]. The conversion rates of CLS for rectal cancer typically ranged from 9 to 16% in large RCTs [2, 5, 6], and can be as high as 25% in patients with rectal cancer receiving NCRT [8]. Although the ROLARR trial failed to indicate the superiority of RALS over CLS in terms of the conversion rate to open laparotomy (8.1% vs 12.2%, *p* = 0.16) [10], several meta-analyses have reported a lower conversion rate for RALS compared with CLS [25, 26, 28, 29]. In our study, no conversion was noted in the RALS group, and only one patient (1.2%) required conversion to open laparotomy due to tumor-related factors in the CLS group in the overall cohort. These results were not uncommon for RALS, but were favorably comparable to the conversion rate of CLS for rectal cancer in the COREAN trial [1]. However, since we have been performing CLS for locally advanced rectal cancer after administering NCRT since 2011, our low conversion rate to laparotomy of CLS for rectal cancer might reflect our abundant and advanced expertise.

Several previous studies have shown that RALS for rectal cancer is associated with a significantly longer operative time compared with CLS for rectal cancer [16, 23, 28, 29]. Inconsistent with the results of previous studies, median operative times were comparable between the groups in our study. Moreover, the present study included the learning curve period, which was previously reported as ranging from 20 to 44 cases [18, 32, 33]. After overcoming this learning curve and acquiring adequate expertise, such as in the working of the camera and manipulation of robotic forceps, and with practice in the set up for the robotic system, our operative time might become shorter than reported here.

Although NCRT is an established risk factor for postoperative complications, the complication rate in our study was comparable to the previously reported rates of 8.9–33.1% and 18.4%-31.7% for RALS and CLS, respectively [10, 12, 17, 18, 24, 27]. Postoperative complication (CD grade ≥ II) rates in the RALS group were significantly lower than those of CLS in this study. The superiority of RALS in terms of postoperative complications could be mainly due to the lower occurrence of anastomotic leakage and urinary retention in the RALS group, because there was a tendency to lower rates of these complications in the RALS compared with the CLS group in both cohorts. The frequency of anastomotic leakage in this study was 15.1% in the overall cohort (RALS 0.0% vs. CLS 20.5%) and 9.1% in the matched cohort (RALS 0.0% vs. CLS 25.0%). Previous studies demonstrated that anastomotic leakage occurred in 1.5–12.2% of patients who underwent RALS and 1.8–10.4% of those who underwent CLS [10, 12, 17, 18, 24, 27]. In our study, although the rates of anastomotic leakage in the RALS group were much lower than those in previous reports, leakage rates in the CLS group were extremely high compared with those in previous reports. To evaluate the risk factors for anastomotic leakage, we performed sub-group analyses in the 53 patients who underwent LAR among the overall cohort (RALS: 14 cases, CLS: 39 cases). Eight of the 53 patients had anastomotic leakage > CD grade II, including six male (6/31; 19.4%) and two female patients (2/22; 9.1%) (*p* = 0.2912); two of the patients (2/6; 33.3%) were older than 75 years and six patients (6/47; 12.8%) were aged less than 75 years (*p* = 0.2298). In terms of intraoperative blood flow assessment with ICG injection, anastomotic leakage occurred in seven patients (7/35; 20.0%) who did not undergo the blood flow test and one patient (1/18; 5.6%) in whom the test was performed (*p* = 0.1355). Recent studies have shown the potential benefits of intraoperative ICG imaging for decreasing anastomotic leakage in MIS for rectal cancer [34, 35]. In the present study, the rates of intraoperative blood flow assessed using ICG were significantly higher for both cohorts in the RALS group compared to the CLS group (*p* < 0.0001). One of the reasons for the significant difference in the incidence of anastomotic leakage between groups could be the significant differences observed in the results of the intraoperative blood flow test with ICG between groups. Thus, although male sex, elderly age, and not performing the blood flow test were associated with slightly higher rates of anastomotic leakage compared with their counterparts, the differences were not significant. In terms of the surgical approach, on the other hand, eight patients (8/39; 20.5%) who underwent CLS and none of the RALS patients developed anastomotic leakage (*p* = 0.0201). In terms of BMI, four patients (4/11; 36.4%) with BMI ≥ 26 kg/m^2^ and four patients (4/42; 9.5%) with BMI < 26 kg/m^2^ developed anastomotic leakage (*p* = 0.0418), suggesting that obesity might be a risk factor for anastomotic leakage in patients undergoing CLS. In the ROLARR trial, the conversion rate to open laparotomy in the obese patients who underwent RALS and CLS was relatively high (10/53; 18.9%, 15/54; 27.8%, respectively) [10]. These results might suggest that the obesity contribute to difficulty of the surgical procedure including anastomosis in MIS.

The frequency of urinary retention in the present study was 4.5% in the overall cohort (RALS 0.0% vs. CLS 6.2%) and 3.3% in the matched cohort (RALS 0.0% vs. CLS 6.7%), showing that the rates of urinary retention in the RALS group tended to be lower than those in the CLS group in both cohorts. Moreover, the days to commencement of a soft diet and duration of postoperative hospital stay were both more favorable in the RALS than the CLS group in both cohorts. Previous studies have reported that RALS offers potential benefits, such as a lower complication rate, shorter postoperative hospital stay, and more favorable functional results, and several factors have been proposed to explain the reason why the robotic approach is more advantageous than the conventional laparoscopic approach for rectal cancer [11, 12, 23, 25–28]. Wristed instruments enable ambidextrous capability and intuitive manipulation by the surgeons, the camera provides stable three-dimensional high-definition imaging, and the robotic arm provides steady retraction and exposure. Combination of these functions in the robotic system facilitates accurate anatomical dissection in the deep and narrow pelvis, and might enable greater preservation of pelvic autonomic functions. The potential advantages of RALS might be more pronounced under specific conditions, such as lower rectal cancer, in male and obese patients, and those undergoing surgery after NCRT [10, 18, 26].

We observed no significant differences in resection margins (proximal and distal), positive RMs, or the number of lymph nodes dissected, between the two groups in both cohorts. Consistent with the above results, analysis of the patients who underwent LAR showed no significant differences in terms of DM between groups in the matched cohorts (RALS: 31.4 ± 15.8 mm, CLS: 28.5 ± 16.1 mm, *p* = 0.6819, data not shown). The technical and oncological results of the procedure in the present study were comparable between the RALS and CLS approaches. This might suggest that the technical and oncological results of the procedure in CLS for rectal cancer are virtually equivalent to those of RALS, when performed by experienced laparoscopic surgeons. However, further studies are necessary to evaluate the other pathological parameters, such as CRM, which is an important predictor of oncological prognosis [36].

Our study has some limitations. The first is its non-randomized and retrospective design. To overcome this limitation, we performed case-matched analysis using several clinical variables. Thus, the groups were well balanced and selection bias was obviated. Second, our cohort study was performed at a single center and was relatively limited. Therefore, the number of cases in our study might be insufficient to draw decisive conclusions. Third, our propensity score-matched analysis mainly took baseline characteristics into account, and did not consider latent confounders, such as surgeon’s experience and the learning curve. Fourth, we did not assess postoperative sexual function, which is essential to evaluate the evidence concerning the clinical benefits of a treatment modality, and we only assessed voiding function without using a questionnaire about voiding function. Finally, we evaluated proximal, distal and radial margins as pathological parameters for assessment of the technical and oncological results of the procedure, and not the CRM. Hence, our pathological analysis might be inadequate for assessing the completeness and quality of TME.

## Conclusion

In conclusion, the present propensity score-matched analysis suggested that RALS for patients with rectal cancer undergoing NCRT is safe and feasible. The results indicated that the operative and pathological outcomes of RALS might be better or similar to those of CLS. Further research is required in the near future to determine whether equal long-term oncological outcomes are observed following the two procedures.

## References

[1] Kang SB, Park JW, Jeong SY (2010). Open versus laparoscopic surgery for mid or low rectal cancer after neoadjuvant chemoradiotherapy (COREAN trial): short-term outcomes of an open-label randomised controlled trial. Lancet Oncol.

[2] van der Pas MH, Haglind E, Cuesta MA, Fürst A, Lacy AM, Hop WC, Bonjer HP COlorectal cancer Laparoscopic or Open Resection II (Color II) Study group (2013) Laparoscopic versus open surgery for rectal cancer (COLOR II): short-term outcomes of a randomised, phase 3 trial. Lancet Oncol 14:210-218. 10.1016/S1470-2045(13)70016-0 10.1016/S1470-2045(13)70016-0 23395398

[3] Jeong SY, Park JW, Nam BH (2014). Open versus laparoscopic surgery for mid-rectal or low-rectal cancer after neoadjuvant chemoradiotherapy (COREAN trial): survival outcomes of an open-label, non-inferiority, randomised controlled trial. Lancet Oncol.

[4] Bonjer HJ, Deijen CL, Abis GA (2015). A randomized trial of laparoscopic versus open surgery for rectal cancer. N Engl J Med.

[5] Fleshman J, Branda M, Sargent DJ (2015). Effect of laparoscopic-assisted resection vs open resection of stage II or III rectal cancer on pathologic outcomes: The ACOSOG Z6051 randomized clinical trial. JAMA.

[6] Stevenson AR, Solomon MJ, Lumley JW, ALaCaRT investigators (2015). Effect of laparoscopic-assisted resection vs open resection on pathological outcomes in rectal cancer: The ALaCaRT randomized clinical trial. JAMA.

[7] Sauer R, Becker H, Hohenberger W, et al, German Rectal Cancer Study Group (2004) Preoperative versus postoperative chemoradiotherapy for rectal cancer. N Engl J Med 351:1731–1740. 10.1056/NEJMoa040694 10.1056/NEJMoa040694 15496622

[8] Denoya P, Wang H, Sands D, Nogueras J, Weiss E, Wexner SD (2010). Short-term outcomes of laparoscopic total mesorectal excision following neoadjuvant chemoradiotherapy. Surg Endosc.

[9] Sato T, Ozawa H, Hatate K (2011). A Phase II trial of neoadjuvant preoperative chemoradiotherapy with S-1 plus irinotecan and radiation in patients with locally advanced rectal cancer: clinical feasibility and response rate. Int J Radiat Oncol Biol Phys.

[10] Jayne D, Pigazzi A, Marshall H (2017). Effect of robotic-assisted vs conventional laparoscopic surgery on risk of conversion to open laparotomy among patients undergoing resection for rectal cancer: the ROLARR randomized clinical trial. JAMA.

[11] Kim JY, Kim NK, Lee KY, Hur H, Min BS, Kim JH (2012). A comparative study of voiding and sexual function after total mesorectal excision with autonomic nerve preservation for rectal cancer: laparoscopic versus robotic surgery. Ann Surg Oncol.

[12] Yamaguchi T, Kinugasa Y, Shiomi A, Tomioka H, Kagawa H, Yamakawa Y (2016). Robotic-assisted vs. conventional laparoscopic surgery for rectal cancer: short-term outcomes at a single center. Surg Today.

[13] Kim HJ, Choi GS, Park JS, Park SY, Yang CS, Lee HJ (2018). The impact of robotic surgery on quality of life, urinary and sexual function following total mesorectal excision for rectal cancer: a propensity score-matched analysis with laparoscopic surgery. Colorectal Dis.

[14] Matsuyama T, Endo H, Yamamoto H, et al (2021) Outcomes of robot-assisted versus conventional laparoscopic low anterior resection in patients with rectal cancer: propensity-matched analysis of the National Clinical Database in Japan. BJS Open 5(5). 10.1093/bjsopen/zrab083 10.1093/bjsopen/zrab083 PMC8458638 34553225

[15] Debakey Y, Zaghloul A, Farag A, Mahmoud A, Elattar I (2018). Robotic-assisted versus conventional laparoscopic approach for rectal cancer surgery, first Egyptian academic center experience. RCT Minim Invasive Surg.

[16] Kim MJ, Park SC, Park JW (2018). Robot-assisted versus laparoscopic surgery for rectal cancer: a phase II open label prospective randomized controlled trial. Ann Surg.

[17] Serin KR, Gultekin FA, Batman B (2015). Robotic versus laparoscopic surgery for mid or low rectal cancer in male patients after neoadjuvant chemoradiation therapy: comparison of short-term outcomes. J Robot Surg.

[18] Huang YM, Huang YJ, Wei PL (2017). Outcomes of robotic versus laparoscopic surgery for mid and low rectal cancer after neoadjuvant chemoradiation therapy and the effect of learning curve. Medicine (Baltimore).

[19] Brierley JD, Gospodarowicz MK, Wittekind C (2017). TNM classification of malignant tumors.

[20] Japanese Society for Cancer of the Colon and Rectum (1998) General rules for clinical and pathological studies on cancer of the colon, rectum, and anus, 6th ed. Kanehara & Co, Tokyo

[21] Sato T, Kokuba Y, Koizumi W, Hayakawa K, Okayasu I, Watanabe M (2007). Phase I trial of neoadjuvant preoperative chemotherapy with S-1 and irinotecan plus radiation in patients with locally advanced rectal cancer. Int J Radiat Oncol Biol Phys.

[22] Dindo D, Demartines N, Clavien PA (2004). Classification of surgical complications: a new proposal with evaluation in a cohort of 6336 patients and results of a survey. Ann Surg.

[23] Kang J, Yoon KJ, Min BS (2013). The impact of robotic surgery for mid and low rectal cancer: a case-matched analysis of a 3-arm comparison–open, laparoscopic, and robotic surgery. Ann Surg.

[24] Cho MS, Baek SJ, Hur H (2015). Short and long-term outcomes of robotic versus laparoscopic total mesorectal excision for rectal cancer: a case-matched retrospective study. Medicine (Baltimore).

[25] Xiong B, Ma L, Huang W, Zhao Q, Cheng Y, Liu J (2015). Robotic versus laparoscopic total mesorectal excision for rectal cancer: a meta-analysis of eight studies. J Gastrointest Surg.

[26] Sun Y, Xu H, Li Z (2016). Robotic versus laparoscopic low anterior resection for rectal cancer: a meta-analysis. World J Surg Oncol.

[27] Law WL, Foo DCC (2017). Comparison of short-term and oncologic outcomes of robotic and laparoscopic resection for mid- and distal rectal cancer. Surg Endosc.

[28] Li X, Wang T, Yao L (2017). The safety and effectiveness of robot-assisted versus laparoscopic TME in patients with rectal cancer: a meta-analysis and systematic review. Medicine (Baltimore).

[29] Prete FP, Pezzolla A, Prete F (2018). Robotic versus laparoscopic minimally invasive surgery for rectal cancer: a systematic review and meta-analysis of randomized controlled trials. Ann Surg.

[30] Chan AC, Poon JT, Fan JK, Lo SH, Law WL (2008). Impact of conversion on the long-term outcome in laparoscopic resection of colorectal cancer. Surg Endosc.

[31] Law WL, Poon JT, Fan JK, Lo SH (2009). Comparison of outcome of open and laparoscopic resection for stage II and stage III rectal cancer. Ann Surg Oncol.

[32] Park EJ, Kim CW, Cho MS (2014). Multidimensional analyses of the learning curve of robotic low anterior resection for rectal cancer: 3-phase learning process comparison. Surg Endosc.

[33] Yamaguchi T, Kinugasa Y, Shiomi A (2015). Learning curve for robotic-assisted surgery for rectal cancer: use of the cumulative sum method. Surg Endosc.

[34] Watanabe J, Ishibe A, Suwa Y (2020). Indocyanine green fluorescence imaging to reduce the risk of anastomotic leakage in laparoscopic low anterior resection for rectal cancer: a propensity score-matched cohort study. Surg Endosc.

[35] Yanagita T, Hara M, Osaga S (2021). Efficacy of intraoperative ICG fluorescence imaging evaluation for preventing anastomotic leakage after left-sided colon or rectal cancer surgery: a propensity score-matched analysis. Surg Endosc.

[36] Kusters M, Marijnen CA, van de Velde CJ (2010). Patterns of local recurrence in rectal cancer; a study of the Dutch TME trial. Eur J Surg Oncol.

